# Repetitive Transcranial Magnetic Stimulation Promotes Neural Stem Cell Proliferation and Differentiation after Intracerebral Hemorrhage in Mice*

**DOI:** 10.1177/0963689719834870

**Published:** 2019-03-04

**Authors:** Mengchu Cui, Hongfei Ge, Han Zeng, Hongxiang Yan, Le Zhang, Hua Feng, Yujie Chen

**Affiliations:** 1 Department of Neurosurgery, Southwest Hospital, Third Military Medical University, Chongqing, P. R. China; 2 College of Computer and Information Science, Southwest University, Chongqing, P. R. China; 3 College of Computer Science, Sichuan University, Chengdu, P. R. China; *This article was originally submitted for publication in the special issue on stroke.

**Keywords:** repetitive transcranial magnetic stimulation, intracerebral hemorrhage, neural stem cell, proliferation, differentiation, mitogen-activated protein kinase

## Abstract

Repetitive transcranial magnetic stimulation (rTMS) is a physical treatment applied during recovery after intracerebral hemorrhage (ICH). With in vivo and in vitro assays, the present study sought to investigate how rTMS influences neural stem cells (NSCs) after ICH and the possible mechanism. Following a collagenase-induced ICH, adult male C57BL/6 J mice were subjected to rTMS treatment every 24 h for 5 days using the following parameters: frequency, 10 Hz; duration, 2 s; wait time, 5.5 s; 960 trains (500 µV/div, 5 ms/div, default setting). Brain water content and neurobehavioral score were assessed at days 1, 3, and 5 after ICH. The proliferation and differentiation of NSCs were observed using immunofluorescence staining for Nestin, Ki-67, DCX, and GFAP on day 3 after ICH, and rTMS treatment with the same parameters was applied to NSCs in vitro. We found that rTMS significantly reduced brain edema and alleviated neural functional deficits. The mice that underwent ICH recovered faster after rTMS treatment, with apparent proliferation and neuronal differentiation of NSCs and attenuation of glial differentiation and GFAP aggregation. Accordingly, proliferation and neuronal differentiation of isolated NSCs were promoted, while glial differentiation was reduced. In addition, microarray analysis, western blotting assays, and calcium imaging were applied to initially investigate the potential mechanism. Bioinformatics showed that the positive effect of rTMS on NSCs after ICH was largely related to the MAPK signaling pathway, which might be a potential hub signaling pathway under the complex effect exerted by rTMS. The results of the microarray data analysis also revealed that Ca^2+^ might be the connection between physical treatment and the MAPK signaling pathway. These predictions were further identified by western blotting analysis and calcium imaging. Taken together, our findings showed that rTMS after ICH exhibited a restorative effect by enhancing the proliferation and neuronal differentiation of NSCs, potentially through the MAPK signaling pathway.

## Introduction

Stroke is a major threat to human health that always results in permanent injuries and causes a series of complications in patients^1^. Intracerebral hemorrhage (ICH) with evident high morbidity and mortality comprises approximately one-third of stroke cases, although its pathophysiologic mechanism remains unclear and effective treatments for stroke are lacking^2^. A clinical trial of extrinsic drugs did not obtain satisfactory results^3^. Intrinsic neural stem cells (NSCs), discovered in 1992, provide a direct way to repair the brain^4^. Activation of proliferation, differentiation, and migration has been reported as the possible major intrinsic protective mechanism after ICH. However, accompanied by limited restoration, a method of influencing the intrinsic and natural repair system in the body demonstrates a promising means of ICH rehabilitation^5^.

Recent research has shown the existence of an intrinsic biological electromagnetic field during the period of development and repair after neuronal damage^6^. Different properties of electromagnetic fields with spatial and temporal differences may exert various influences on NSCs, especially on their proliferation, differentiation, and migration^7^.

Repetitive transcranial magnetic stimulation (rTMS), which is widely used in neural diseases, is a physical method that can noninvasively penetrate the scalp to exert a focal electromagnetic field on neural cells^8^. Though effects of this noninvasive treatment are substantial, little research has focused on the influence of rTMS on NSCs in ICH since its invention in 1985. In recent reports, researchers showed that rTMS could promote the proliferation of NSCs in ischemic rats^9–11^. Nevertheless, no research has focused on the effects and potential underlying mechanism of rTMS on ICH.

This study was designed to examine the influence of rTMS on neurological function, cerebral edema, glial aggregation, and the migration, proliferation, and neuronal differentiation of NSCs in a mouse model of experimental ICH. In addition, the potential mechanisms underlying the alterations in NSCs triggered in vitro by the treatment of rTMS were investigated and discussed in this study.

## Materials and Methods

### Ethics Statement

This study was approved by the Ethic Committee of Southwest Hospital, Chongqing, China. All animals were provided by the animal center of the Third Military Medical University, Chongqing, China. The experiment was designed and performed in strict conformity with the Guide for the Care and Use of Laboratory Animals of NIH (NIH Pub. No. 85-23, revised 1996) and the 3 R principle; we have done our best to minimize the number of animals included and their suffering.

### Experimental Animals and Groups

Seven- to eight-week-old male C57BL/6 J mice, weighing 23–25 g, were used in the experiment. They were randomly classified into three groups: the sham group (*n*=15), the ICH group (*n*=15), and the ICH/rTMS group (*n*=15). All mice were housed in a laminar flow room, given clean food and water, provided with enough room, and cared for by designated technicians.

### Experimental ICH Model Protocol

The mice were anesthetized intraperitoneally with 5% chloral hydrate (7 ml/kg, Sigma-Aldrich, St. Louis, MO, USA) and placed in a stereotaxic apparatus (Stoelting, Kiel, WI, USA). With the mouse skull revealed and bregma exposed, a 1-mm cranial burr hole (coordinates: 2.0 mm lateral to the midline, 1.0 mm posterior to the bregma) was made with a cranial drill. Collagenase type VII solution (0.3 µl, 0.1 µg in 1 ml of saline) in a 5 µl micro-syringe (Hamilton Bonaduz AG, Shanghai, China) was injected into the right basal ganglia region (3 mm deep from the dura mater) through a micro-infusion pump (Harvard Apparatus, Holliston, MA, USA) at a rate of 0.3 µl/min. To allow time for the mouse to adapt and to avoid reflux, the needle of the micro-syringe was required to be kept in place for 5 min after the injection, after which it was carefully withdrawn. During the period of modeling and recovery from anesthesia, mice were placed on a heating pad to maintain their body temperature in the range of 36.5–37.5°C. The sham group received the same procedures except they were injected with saline.

### Neurobehavioral Evaluation

Using the guidance of neurological severity scores for mice, which assess balance, reflexes, and movement, and the sensitivity to sensation in all groups, neurobehavioral evaluation was carried out by two investigators who were blinded to the grouping of the mice. The evaluation scale ranges from 0 to 18 with normal observations marked 18 and the maximal deficit considered 0. The observation points were set at 0 h, 24 h, 72 h, and 120 h after the operation. In cases of deviation, only those (except the sham group) marked between 5 and 9 at the 0 h time point after the surgery were included^10^. The 0 h neurobehavioral assessment was performed just after the mice recovered from surgical anesthetization.

### In vivo rTMS Treatment

**Fig. 1.**
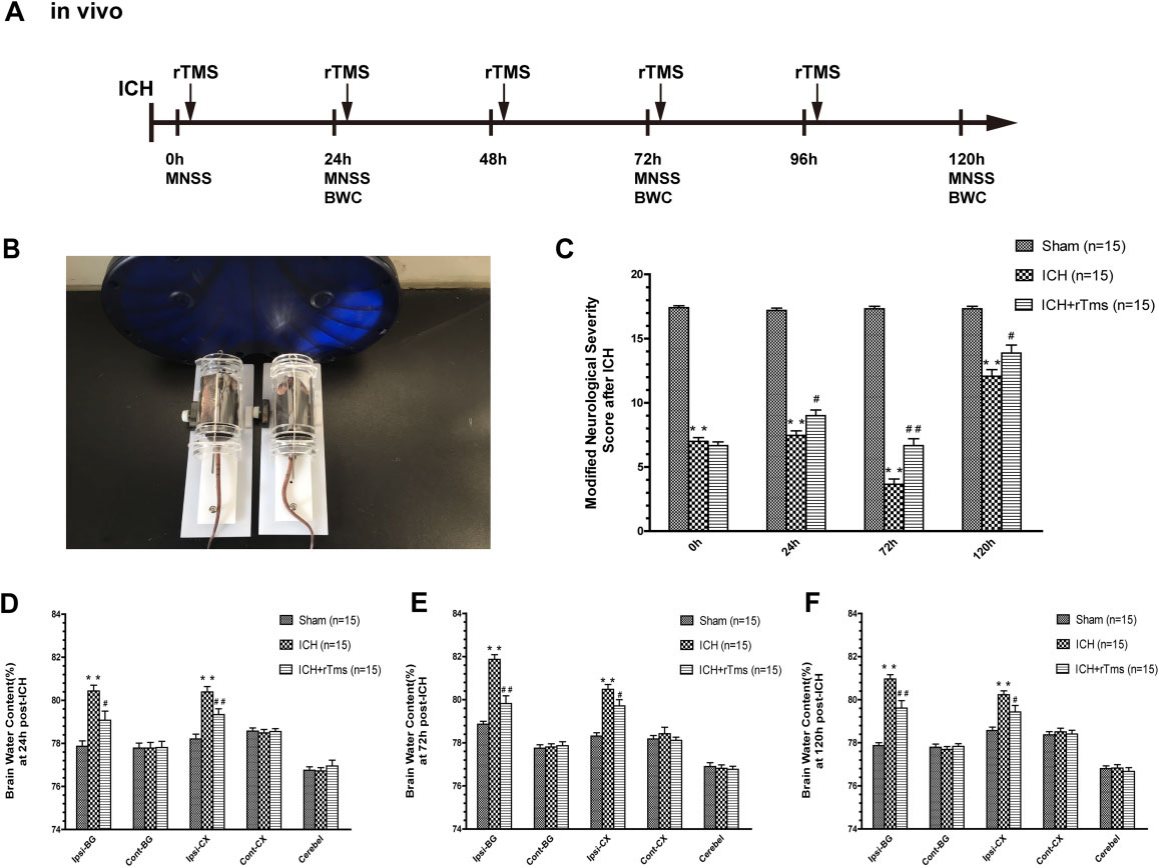
The effects of rTMS on the recovery of neurological function and brain edema. (A) Scheme and schedule of the in vivo experiment; (B) in vivo rTMS of NSCs; (C) Modified Neurological Severity score (MNSS) in the sham, ICH, and ICH+rTMS groups at 0 h, 24 h, 72 h and 120 h after ICH; Brain water content assessment (BWC) at (D) 24 h, (E) 72 h and (F) 120 h after ICH in the sham, ICH, and ICH+rTMS groups. *n* = 15 for the Modified Neurological Severity score; *n* = 15 for brain water content assessment; *p < 0.05 vs. sham; **p < 0.01 vs. sham; ^#^*p* < 0.05 vs. ICH; ^##^*p* < 0.01 vs. ICH.

Unanesthetized mice were secured into a specialized device (a plastic cylinder that could be penetrated by a magnetic field) that restricted their movement, and rTMS was performed using a Magstim device (RAPID2 model, the Magstim Co, Carmarthenshire, UK) with a figure-eight coil (Fig. 1b). After the 0 h neurobehavioral assessment was performed, all groups received rTMS every 24 h for 5 days with the following parameters: frequency, 10 Hz; duration, 2 s; wait time, 5.5 s; 960 trains (500 µV/div, 5 ms/div, default setting)^9^ ^–11^ (Fig. 1a). The front of the coil was placed above and close to the head in a horizontal position, while the other two groups were in a vertical position. For the purpose of reducing any possible deviation, the mice were gradually acclimated to the plastic cylinder and the noise, rather than magnetic stimuli, made by the device 1 h a day for 1 week before the surgery^12^.

### Determination of Brain Water Content

To determine the brain water content, deeply anesthetized animals were euthanized and decapitated at 24 h, 72 h, and 120 h after the rTMS treatment. Then, the brain was divided into the contralateral cortex (Cont-CX) and basal ganglia (Cont-BG), ipsilateral cortex (Ipsi-CX), and basal ganglia (Ipsi-BG), and cerebellum and the wet weight was measured as soon as possible. All the specimens were incubated in a 105°C heating oven for 72 h and the dry weight was quickly assessed. The formula (wet weight – dry weight)/wet weight × 100% was used to calculate the brain water content as a percentage.

### Tissue Preparation

Seventy-two hours after the rTMS treatment, transcranial perfusion was performed on deeply anesthetized mice with saline and 4% paraformaldehyde (PFA). The brain was removed and postfixed in 4% PFA at 4°C overnight, followed by immersion in 30% PFA-sucrose (30 g sucrose in 70 ml 4% PFA) for dehydration and further fixation at 4°C overnight. To obtain frozen sections for staining, the fixed brains were sliced into 20 μm sections by a cryostat microtome (Leica, Buffalo Grove, IL USA). The frozen sections were washed with 0.1 M phosphate-buffered saline (PBS) and stored in cryoprotectant solution in a 6-well plate at –20°C.

### Immunofluorescence Staining and Image Processing

The stored frozen sections were rinsed in 0.1 M PBS (3 times for 5 min each time) at first, permeabilized with 0.5% Triton X-100 (Beyotime, Shanghai, China) and incubated with 3% bovine serum albumin (BSA, Boster, Wuhan, China) at room temperature. The sections were incubated at 4°C overnight with the following primary antibodies: rabbit polyclonal anti-Ki-67 antibody (1:200, Abcam, Cambridge, MA, USA), rabbit polyclonal anti-doublecortin (DCX) antibody (1:100, Abcam), rabbit polyclonal anti-glial fibrillary acidic protein (GFAP) antibody (1:500, Abcam), and mouse monoclonal anti-Nestin antibody (1:100, Abcam). Afterward, the sections were incubated in the corresponding secondary antibodies, goat anti-rabbit IgG (Alexa Fluor 555, Invitrogen, Carlsbad, CA, USA) or goat anti-mouse IgG (Alexa Fluor 488, Invitrogen), at 37°C for 1 h, and 4′,6-diamidino-2-phenylindole (DAPI) was used to stain nuclei at room temperature for 10 min. Slides were made for observation and storage. Immunofluorescence was examined by confocal microscopy (LSM800, ZEISS, Oberkochen, Germany), and images were obtained using an LSM Image Examiner. The numbers of Nestin-, DCX-, GFAP-, and Ki-67-positive cells were counted using ImageJ (National Institutes of Health, Bethesda, MD, USA).

### Extraction and Passage of NSCs (Primary Culture of NSCs)

Fifteen-day pregnant mice were deeply anesthetized with 4% isoflurane, and cervical dislocation was performed. A fetal mouse was obtained after opening the uterus. Immature cortical cells were harvested from the frontal lobe and digested in TrypLE™ Express (5 ml, 1×, Gibco Company, Shanghai, China) at 37°C for 12 min after washing with 0.1 M PBS and DMEM (high-glucose culture medium, Gibco Company). The activity of TrypLE™ Express was quenched by dilution with DMEM. Then, TrypLE™ Express in DMEM was removed, and complete neural stem cell culture medium (5 ml, supplemented with 2% B27; basic fibroblast growth factor, bFGF 20 ng/ml; and epidermal growth factor, EGF 20 ng/ml) was added. Afterward, neurons were resuspended by pipetting. A 200 mesh screen was used to separate the cell medium and the tissue. Cells were incubated in T25 flasks in a cell-specific homothermal (37°C) humidified incubator with a CO_2_ content of 5%. Every 3 days, the cell solution was centrifuged at 200 rpm for 5 min, the supernatant was discarded, and the remaining pellet was disassociated with accutase treatment (Thermo Fisher Scientific, Shanghai, China) for 8 min at room temperature. Centrifugation at 800 rpm for 5 min was required to stop the accutase reaction as well as sediment the single cells. Finally, the sediment was resuspended in complete neurosphere culture medium.

### Identification of NSCs

All the neurospheres were first observed under a light microscope (IX71, Olympus Corporation, Tokyo, Japan) for a morphological examination. Immunofluorescence was used for identification. After several passages, purified neurospheres were allowed to adhere to a poly-L-ornithine-coated confocal dish in the cell incubator overnight. Then, the cells were prefixed with 4% PFA at room temperature for 15–20 min. After several washes in PBS, the neurospheres were treated as previously described. The primary antibodies used in this procedure were as follows: rabbit polyclonal anti-Sox2 antibody (1:200, Invitrogen) and mouse monoclonal anti-Nestin antibody (1:100, Abcam). Goat anti-rabbit IgG (Alexa Fluor 555, Invitrogen) and goat anti-mouse IgG (Alexa Fluor 488, Invitrogen) were used as secondary antibodies.

### In Vitro RTMS Treatment

**Fig. 2.**
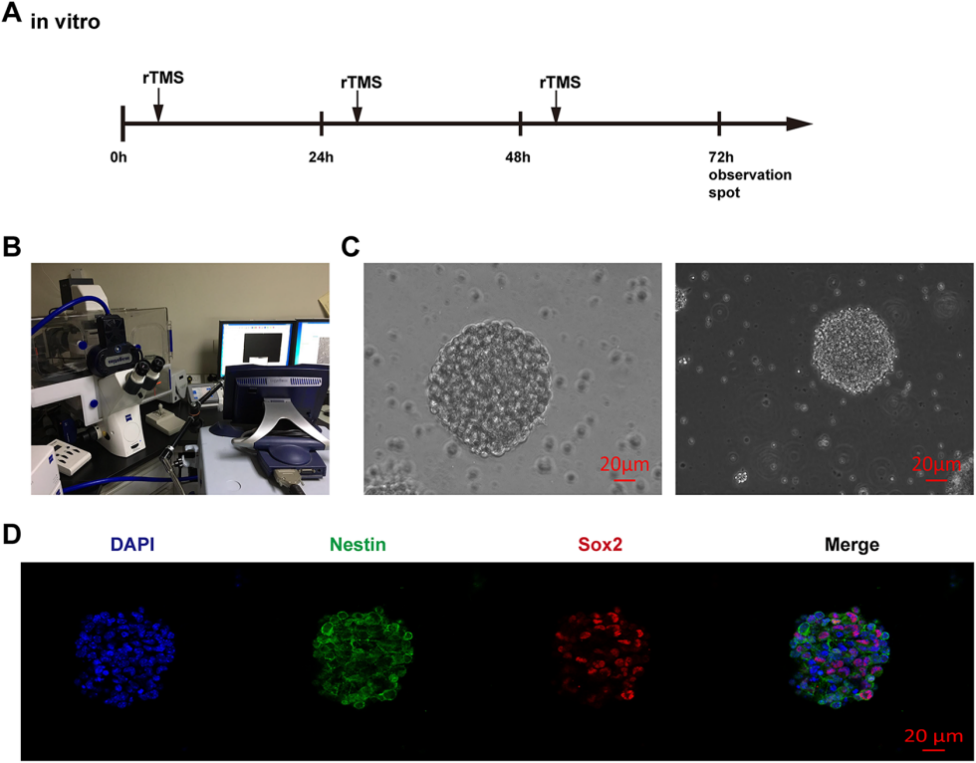
Identification of NSCs. (A) Scheme and schedule of the in vitro experiment; (B) in vitro rTMS of NSCs; (C) Neurospheres observed under light microscopy. Scale bar = 20 μm; (D) immunocharacterization of NSCs, red: SOX2, green: Nestin, blue: DAPI, Scale bar = 20 μm.

After several passages, purified neurospheres were plated in a 24-well plate containing an appropriate poly-L-ornithine-coated coverslip in each well. All wells were supplied with an appropriate volume of complete culture medium. Plates were divided into two groups: the control group and the rTMS group. Cells in the rTMS group were incubated in a small plastic cell incubator with a time-lapse microscope that allowed the magnetic field to penetrate easily, and rTMS was performed using a Magstim device (RAPID2 model, the Magstim Co) with a figure-eight coil, the front of which was closely placed above the plastic incubator in a horizontal position (see Fig. 2b). rTMS was performed every 24 h for 72 h with the following parameters: frequency, 10 Hz; duration, 2 s; wait time, 5.5 s; 960 trains (500 µV/div, 5 ms/div, default setting) (Fig. 2a). The control group was maintained in a standard metal cell incubator that can naturally block any exterior magnetic field. For the differentiation part of this experiment, we applied 1% fetal bovine serum to simulate an induced environment of differentiation after ICH before the in vitro treatment^12^.

### Light Microscopic Observation and Image Processing

After the rTMS protocol was performed, the coverslips were first examined under a light microscope (IX71, Olympus Corporation) to observe and evaluate the number and size of the neurospheres per group. Briefly, beginning from the top left corner and ending at the bottom right corner, each field on the coverslip was thoroughly examined by a researcher blinded to the group condition, and neurospheres with a size of approximately 50 μm or larger in diameter were included. The mean diameter of neurospheres was presented as the neurosphere-forming frequency (proliferation). Image-Pro Plus 6.0 software (Media Cybernetics, Rockville, MD, USA) was used to process the images for size determination and measurement.

### Immunofluorescence Staining and Image Processing

After the rTMS protocol was performed, the coverslips were treated as previously described, and the primary antibodies used in this procedure were as follows: rabbit polyclonal anti-Ki-67 antibody (1:200, Abcam), rabbit polyclonal anti-doublecortin (DCX) antibody (1:100, Abcam), rabbit polyclonal anti-Sox2 antibody (1:200, Invitrogen), mouse monoclonal anti-GFAP antibody (1:500, Abcam), and mouse monoclonal anti-Nestin antibody (1:100, Abcam). Goat anti-rabbit IgG (Alexa Fluor 555, Invitrogen) and goat anti-mouse IgG (Alexa Fluor 488, Invitrogen) were used as secondary antibodies. The numbers of Sox2-, DCX-, GFAP-, and Ki67-positive cells were counted by ImageJ (National Institutes of Health).

### Microarray Analysis

NSCs from the rTMS group and control group were processed as previously described (*n*=5 per group). According to the manufacturer’s instructions, TRIzol reagent (TaKaRa-Bio, Shiga, Japan) was used to extract total cellular RNA. The purity of the prepared RNA was examined by agarose gel electrophoresis. Microarray hybridization and cDNA labeling using the Affymetrix GeneChip Mouse Gene 1.0ST array platform were performed by Gminix Information Technology Corporation, Shanghai, China. To identify possible related genes and pathways, original microarray data were analyzed by the online analysis tool Gene Cloud of Biotechnology Information (GCBI Platform, Shanghai, China) (www.gcbi.com.cn) based on the Kyoto Encyclopedia of Genes and Genomes (KEGG) Pathway Database and Gene Ontology.

### Western Blotting Analysis and Inhibition of Phosphorylation of Erk and P38

NSCs were rinsed twice with cold PBS and then lysed using protein extraction reagent (Thermo Fisher Scientific), which contains 1% protease inhibitors (Thermo Fisher Scientific). The lysate was centrifuged at 4°C at 16,000 × *g* for 15 min, and proteins were separated with 10% SDS-PAGE and transferred to PVDF (polyvinylidene difluoride) membranes (MilliporeSigma, Burlington, MA, USA). Membranes were blocked with 5% milk solution (Boster) for 2 h and incubated at 4°C overnight with primary antibodies (rabbit polyclonal anti-ERK1/2 antibody, rabbit polyclonal anti-p-ERK1/2 antibody, rabbit polyclonal anti-p38 antibody, rabbit polyclonal anti-p-p38 antibody, rabbit polyclonal anti-SAPK/JNK antibody, rabbit polyclonal anti-p-SAPK/JNK antibody, 1:1000, Cell Signaling Technology, Danvers, MA, USA). Then, the membranes were washed with PBST and incubated with HRP-conjugated secondary antibodies (1:5000, Cell Signaling Technology) for 1 h. Proteins were visualized with Super ECL plus western blotting (Bioground Biotechnology, Chongqing, China) and detected by a ChemiDoc XRS system (Bio-Rad, Hercules, CA, USA). The following reagents were added to the cells for inhibition of phosphorylation: SCH772984 (2 μM, 30 min, SelleckChem, Houston, TX, USA), a specific inhibitor of ERK1/2 phosphorylation, and SB203580 (10 μM, 30 min, SelleckChem), a specific inhibitor of p38 phosphorylation. Protein bands were quantified by volume tools with Image Lab software (Bio-Rad). The results are expressed as the relative expression level, which was normalized to the thinnest band of group.

### Calcium Imaging and Blocking of Ca^2+^ Influx

Calcium imaging was performed following the Fluo-4 calcium imaging kit protocol (Invitrogen). Briefly, medium was removed from NSCs by centrifugation. Cells were washed with Living Cell Imaging Solution (Invitrogen) and stained by incubation in Fluo-4 AM staining solution at 37°C and room temperature for 30 min. After removing Fluo-4 AM staining solution, cells were washed with Living Cell Imaging Solution and treated with 20 mM glucose stock in Living Cell Imaging Solution. Calcium imaging was observed and recorded under a confocal microscope (SP8, Leica). After recording for a short period, control cells treated with ethanol were exposed to rTMS. In the blocking experiment, a selective L-type channel blocker, nifedipine (5 μM, dissolved in ethanol, Sigma-Aldrich, Shanghai, China), was added.

### Statistical Analysis

All data are shown as the mean± SEM and were analyzed using SPSS 17.0 (IBM Corporation, Somers, NY, USA). Statistical comparisons in vitro were performed using an unpaired *t*-test, and one-way ANOVA was used for other analyses. A *p*-value < 0.05 was considered indicative of statistical significance, and Graph Pad Prism was used to generate graphs.

## Results

### rTMS Reduced Brain Water Content and Neurological Function Deficit After ICH

To learn about how rTMS plays an important role in recovery from ICH, we first assessed neurological function deficits. The single-blind method was employed for assessments at 0 h, 24 h, 72 h, and 120 h after the surgery. Compared with the sham group, the group that underwent ICH suffered a worse neurobehavioral deficit at all time points (*p* < 0.05). rTMS treatment improved the neural function of ICH mice after one, two, and three treatments (24 h: ICH group, 7.47 ± 0.35 vs. ICH/rTMS group, 9.00 ± 0.45, *p* = 0.012, F factor = 232.8; 72 h: ICH group, 3.67 ± 0.40 vs. ICH/rTMS group, 6.67 ± 0.53, *p* = 0.0001, F factor = 325.0; 120 h: ICH group, 12.07 ± 0.51 vs. ICH/rTMS group, 13.87 ± 0.62, *p* = 0.034, F factor = 31.35, Fig. 1c), and mice exhibited the most improvement on the third day after ICH.

Because brain edema is considered a main pathological feature for evaluating the severity of brain damage and inflammation, we also used the determination of brain water content to measure the percent change in brain water content in various parts of the brain at 24 h, 72 h, and 120 h after the surgery. Only the ipsilateral basal ganglia (ips-BG) and cortex brain (ips-CX) water content was increased in the rTMS groups compared with those in the sham group (*p* < 0.05, Fig. 1d–f). The rTMS treatment relieved the swelling after one, two, and three treatments (ips-BG: 24 h, ICH group, 80.44 ± 0.26% vs. ICH/rTMS group, 79.08 ± 0.42%, *p* = 0.010, F factor = 16.38; 72 h: ICH group, 81.87 ± 0.22% vs. ICH/rTMS group, 79.82 ± 0.35%, *p* < 0.0001, F factor = 63.04; 120 h: ICH group, 80.96 ± 0.20 vs. ICH/rTMS group, 79.61 ± 0.79%, *p* = 0.0023, F factor = 40.06; ips-CX: 24 h, ICH group, 80.39 ± 0.2%5 vs. ICH/rTMS group, 79.34 ± 0.27%, *p* = 0.0083, F factor = 19.34; 72 h: ICH group, 80.48 ± 0.23% vs. ICH/rTMS group, 79.71± 0.29%, *p* = 0.044, F factor = 22.73; 120 h: ICH group, 80.23 ± 0.18% vs. ICH/rTMS group, 79.43 ± 0.31%, *p* = 0.034, F factor = 13.75). In accordance with the neurological evaluation, mice had a better recover on the third day after the surgery.

### rTMS Enhanced the Proliferation of Intrinsic NSCs after ICH (Ki-67)

**Fig. 3.**
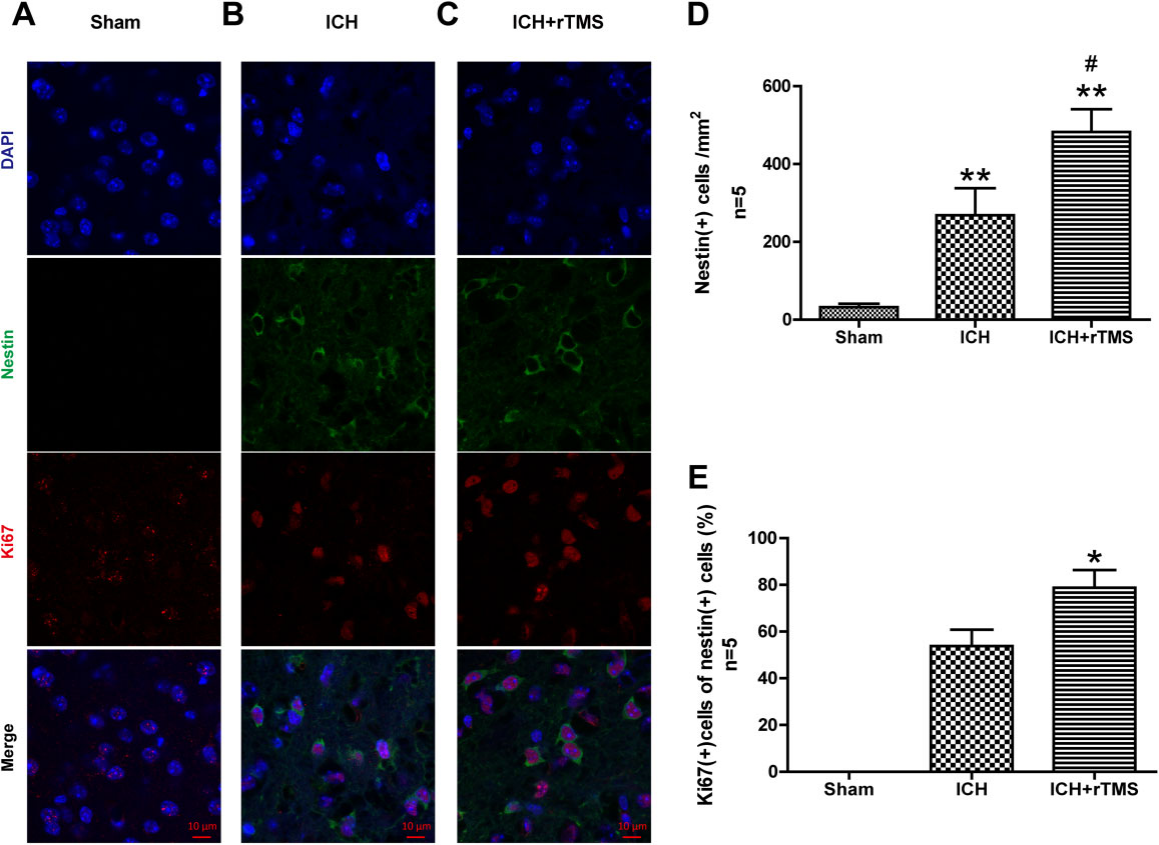
The effects of rTMS on the proliferation of NSCs after ICH. Representative photographs of immunofluorescence co-staining of NSCs for DAPI (blue), Nestin (green), and Ki-67 (red) in (A) sham, (B) ICH, and (C) ICH+rTMS groups at 72 h after ICH. Quantitative analyses of (D) Nestin-positive cells and (E) Ki-67/Nestin double-positive cells in the sham, ICH, and ICH+rTMS groups at 72 h after ICH. *n* = 5 for immunofluorescence staining analysis. Scale bar = 10 μm; *p < 0.05 vs. sham; **p < 0.01 vs. sham; ^#^*p* < 0.05 vs. ICH.

The Ki-67 protein (also known as MKI67) is a cellular marker for cell proliferation. During interphase, the Ki-67 antigen can be specifically detected in the cell nucleus; hence, Ki-67 immunofluorescence labeling was used to evaluate the proliferation of NSCs, which is a natural protection and recovery mechanism after ICH. Immunofluorescence staining demonstrated that the percentage of Ki-67-positive cells in Nestin-positive cells in the perihematomal region (basal ganglia) significantly increased at 72 h after ICH (Fig. 3a–c). In addition, the percentage in the ICH/rTMS group increased almost 46% compared with that in the ICH group after the 72 h rTMS treatment. (53.8±6.9 vs. 78.8±7.6, *p* = 0.041, Fig. 3d, e).

### rTMS Guided Intrinsic NSCs to Differentiate Into Neurons (Reconstruct the Neural Function) after ICH (DCX)

**Fig. 4.**
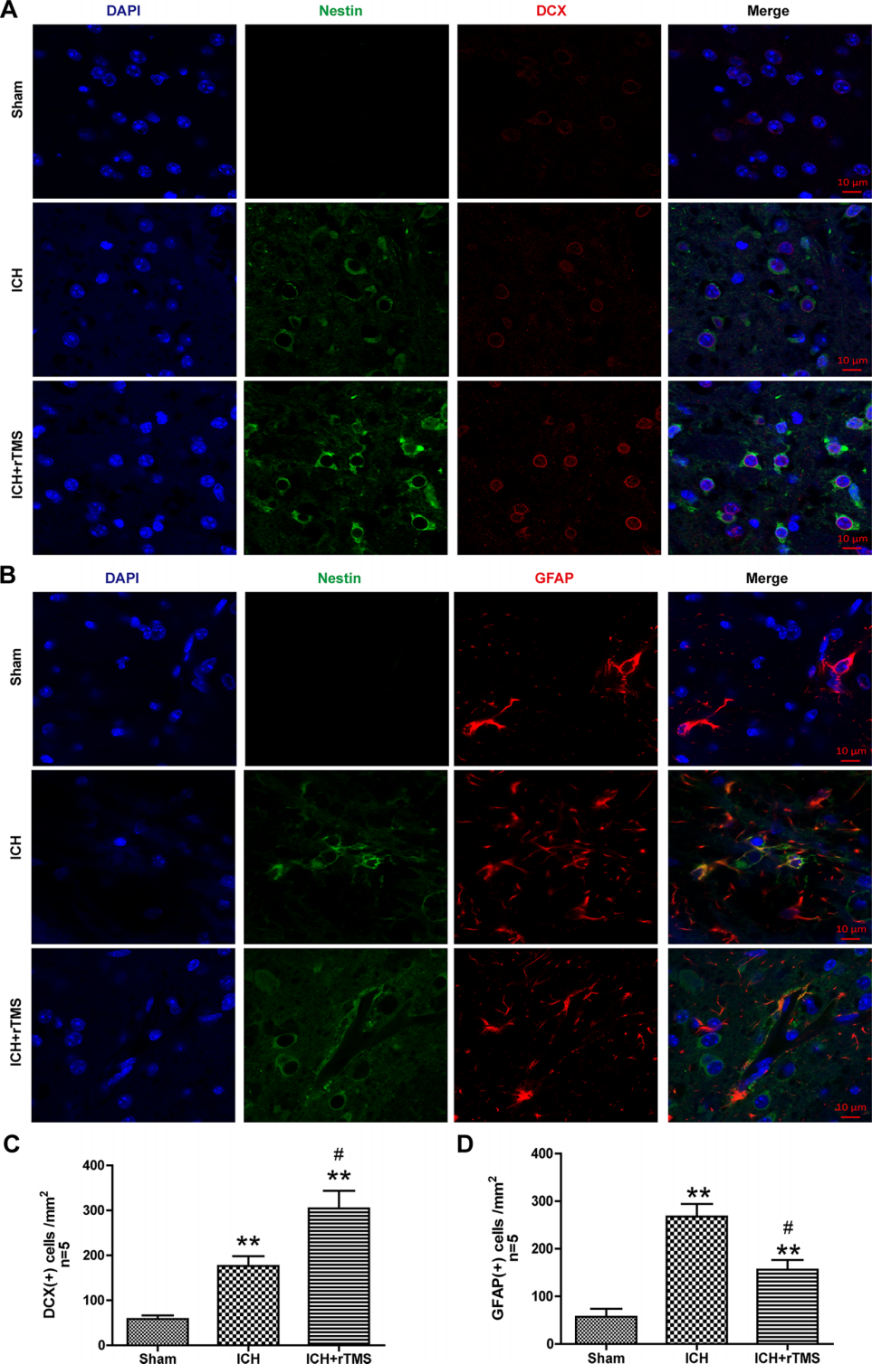
The effects of rTMS on the differentiation of NSCs after ICH. (A) Representative photographs of immunofluorescence co-staining of NSCs for DAPI (blue), Nestin (green) and DCX (red) and (B) immunofluorescence co-staining of NSCs for DAPI (blue), Nestin (green) and GFAP (red) in the sham, ICH, and ICH+rTMS groups at 72 h after ICH. (C) Quantitative analyses of DCX-positive and (D) GFAP-positive cells in the sham, ICH, and ICH+rTMS groups at 72 h after ICH. *n* = 5 for immunofluorescence staining analysis. Scale bar = 10 μm; **p < 0.01 vs. sham; ^#^*p* < 0.05 vs. ICH.

The prognosis of ICH is mainly dependent on the renewal of functional neurons, which reconstruct the serviceable connections among the cells in the central nervous system; glial scars, which consist of glial cells, are the regular pathological course that results in a poor outcome. Whether the rTMS treatments play an important role in recovery from ICH primarily lies in the direction of terminal differentiation of intrinsic NSCs. Doublecortin is a microtubule-associated protein expressed by neuronal precursor cells and immature neurons in embryonic and adult cortical structures. Neuronal precursor cells begin to express DCX while actively dividing, and their neuronal daughter cells continue to express DCX for 2–3 weeks as the cells mature into neurons. DCX immunofluorescence labeling was used in the experiment to determine whether rTMS treatment can promote neuronal repair after ICH (Fig. 4a). The results showed that a few DCX-positive cells emerged after ICH and increased significantly by 72% in the ICH+rTMS group compared with those in the ICH group (176.4±21.8 vs. 304.2±39.4, *p* = 0.022, Fig. 4c).

### rTMS Reduced the Aggregation of Glial Cells After ICH

Aggregation of glial cells, which is treated as a process of inflammatory response in the central nervous system, occurs after brain damage. GFAP is an intermediate filament protein that is expressed by numerous cell types of the central nervous system, especially glial cells. GFAP immunofluorescence labeling was used to detect inflammatory glial cells after ICH (Fig. 4b). The results showed that the number of GFAP-positive cells significantly increased around the hematoma at 72 h after ICH (*p* < 0.05), while after the 72 h rTMS treatment, GFAP-positive cells showed a marked decrease of approximately 41% (267.6±26.5 vs. 156.2±20.3, *p* =0.010, Fig. 4d).

### Identification of NSCs

The isolated NSCs were refined after approximately 2–3 passages, and most impurities and other types of cells were removed. Under a light microscope, shiny and smooth passaged neurospheres were observed, which is an indicator of high cellular activity (Fig. 2c). Afterwards, confocal imaging revealed co-staining of Sox2 (marker of stemness), Nestin (specific marker of NSCs or neural precursor cells) and DAPI, strongly indicating the characteristics of NSCs (Fig. 2d).

### rTMS Promoted the Proliferation of NSCs In Vitro

**Fig. 5.**
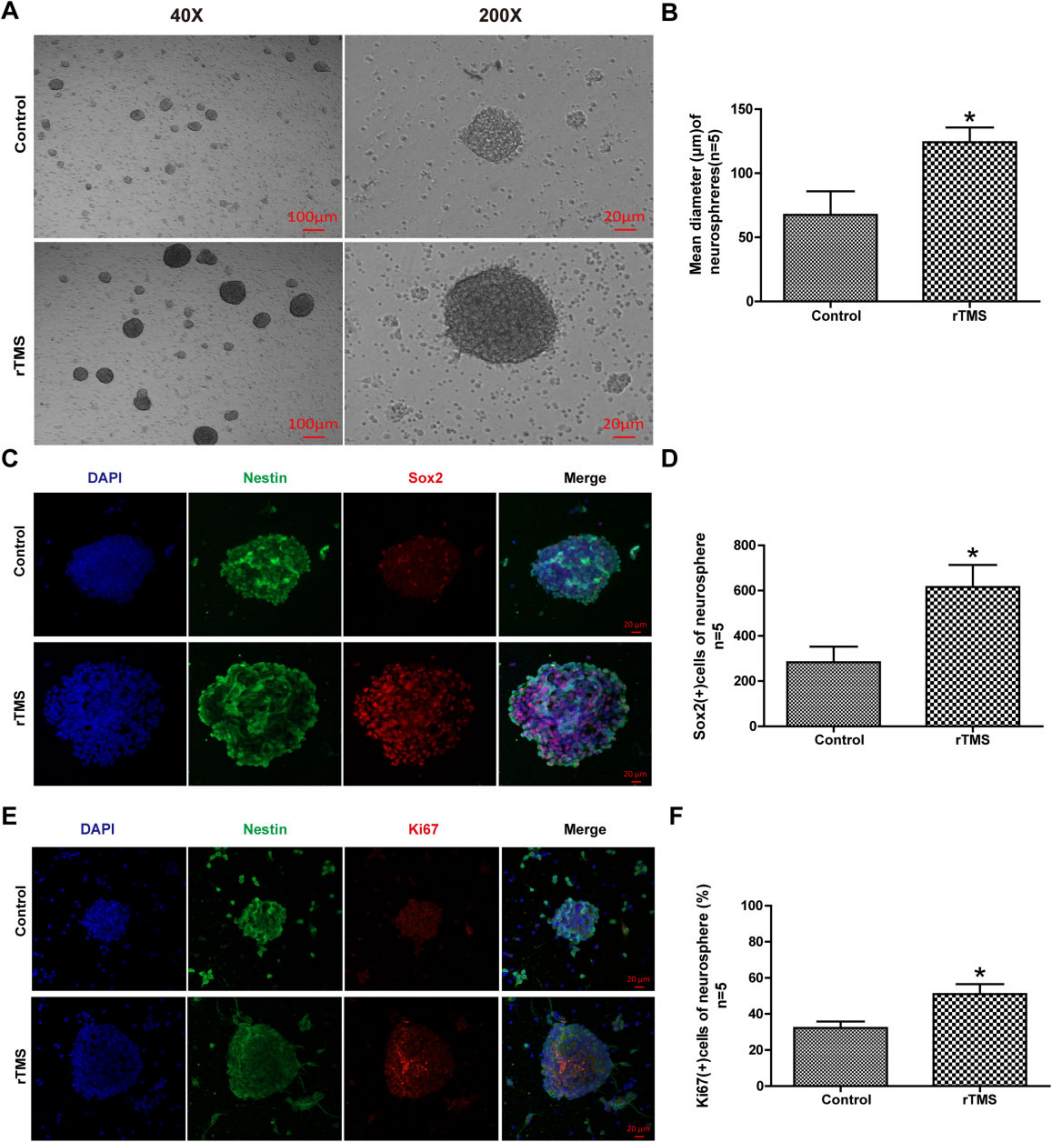
The effects of rTMS on the proliferation of NSCs in vitro. (A) Representative photographs of light microscopy and quantitative analyses of (B) mean diameter of neurospheres in the control and rTMS groups. (C) Representative photographs of immunofluorescence co-staining of NSCs for DAPI (blue), Nestin (green), and Ki-67 (red) in the control and rTMS groups. Quantitative analyses of (D) Ki-67-positive cells and E: Sox2-positive cells in neurospheres in the control and rTMS groups. *n* = 5 for mean diameter analysis, *n* = 5 for immunofluorescence staining analysis. Scale bar = 20 μm; *p < 0.05 vs. control.

After rTMS ---treatment, the neurospheres were examined under a light microscope (Fig. 5a), which showed a notable increase of 83% in the diameter of neurospheres after rTMS exposure (67.6±18.3 vs. 124.2±11.5, *p* = 0.031, Fig. 5b).

SRY-box 2, also known as SOX2, is a transcription factor that is essential for maintaining self-renewal, or pluripotency, of undifferentiated embryonic stem cells. Sox2 plays a critical role in the maintenance of embryonic and neural stem cells. Sox2 and Ki-67 immunofluorescence labeling was used in this experiment to examine the proliferation of NSCs in vitro (Fig. 5c, e). The result demonstrated a significant increase in the number of Sox2-positive cells (117%, 283.0±69.6 vs. 615.4±98.0, *p* =0.024, Fig. 5d) and in the percentage of Ki-67-positive cells per neurosphere compared with those in the control group. (58%, 32.2±3.6 vs. 51.0±5.6, *p* =0.022, Fig. 5f).

### rTMS Modulated the Differentiation of NSCs in vitro

**Fig. 6.**
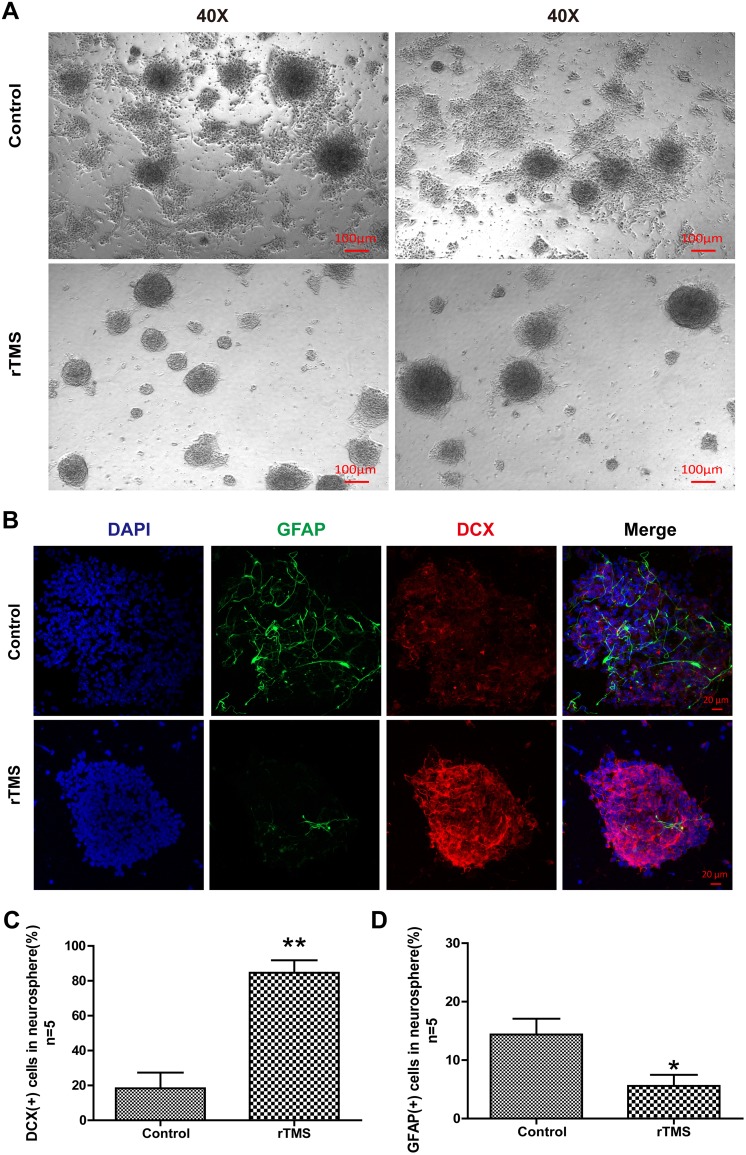
The effects of rTMS on the differentiation of NSCs in vitro. (A) Representative photographs of light microscopy in the control and rTMS groups. Scale bar = 100 μm; (B) Representative photographs of immunofluorescence co-staining of NSCs for DAPI (blue), GFAP (green), and DCX (red) in the control and rTMS groups. Quantitative analyses of (C) DCX-positive cells and (D) GFAP-positive cells in neurospheres in the control and rTMS groups. *n* = 5 for immunofluorescence staining analysis. Scale bar = 20 μm; *p < 0.05 vs. control. **p < 0.01 vs. control.

Neurons and glial cells are the main types of cells derived from NSCs and are the major cell types in the nervous central system. These cells are involved in the recovery from ICH, during which they play critical roles. In brief, neurons and glia decide the outcome of ICH. Under light microscopy, the rTMS group NSCs seldom exhibited adherence and had fewer branches compared with the control group NSCs (Fig. 6a). Based on this finding, NSCs were co-stained for their representative markers DCX and GFAP to examine the differentiation of NSCs in vitro (Fig. 6b). Confocal imaging indicated that the percentage of DCX in every neurosphere in the rTMS group increased significantly compared with that in the control group (360%, 18.4±8.9 vs. 84.6±7.2, *p* = 0.0004, Fig. 6c); in contrast, there was a remarkable reduction in GFAP (82%, 14.4±2.7 vs. 5.6±1.9, *p* = 0.028, Fig. 6d).

### Interaction Network Analysis of rTMS-Related Pathways

**Fig. 7.**
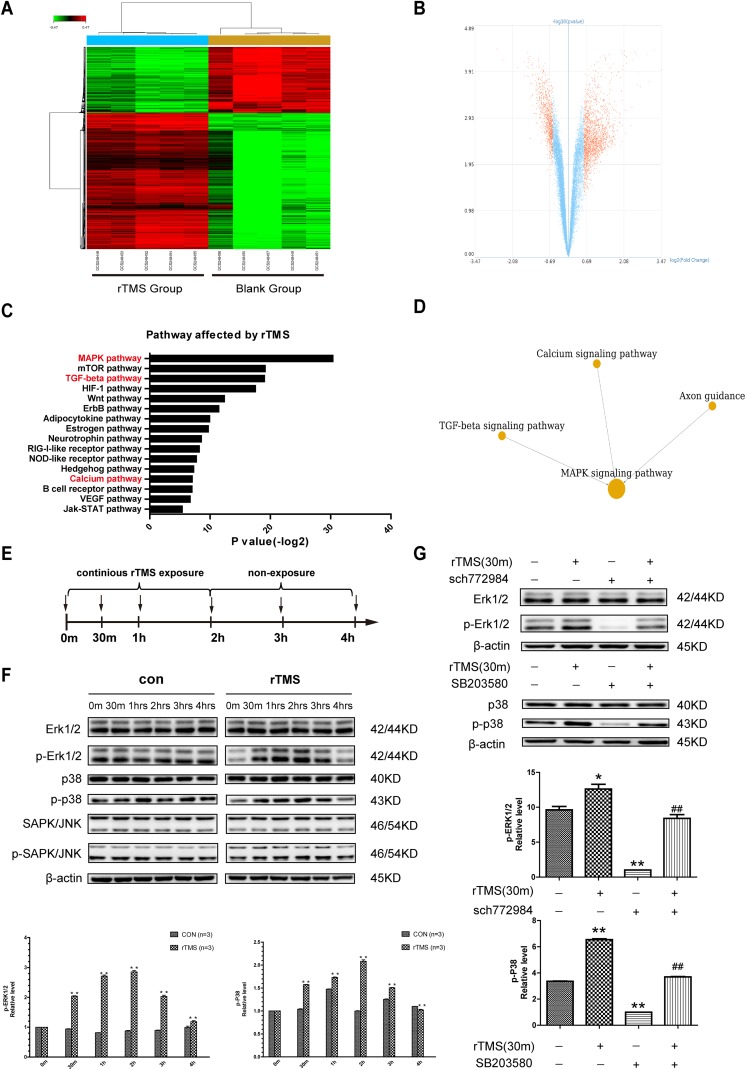
Transcriptome profiling of NSCs in the rTMS group compared with those in the control group indicated the MAPK signaling pathway. (A) Heat map of the differentially expressed genes in five specimens of NSCs from the rTMS group compared with the control group. Red color indicates upregulated genes, and green color indicates downregulated genes (fold change>1.5). (B) Volcano plot of all genes. The −log (P) of each gene is plotted against the log2-fold change (ratio of rTMS group intensity to control group intensity). Red dots indicate genes that were altered after rTMS: the left cluster indicates a downregulation of these genes, while the right one shows an upregulation. (C) Kyoto Encyclopedia of Genes and Genomes pathway enrichment analysis based on distinctly expressed genes between the rTMS-treated NSCs and control NSCs. (D) Path-map network analysis using the GCBI platform revealed that the calcium signaling pathway, TGF-beta signaling pathway, and axon guidance were linked with neurogenesis via MAPK signaling pathways. (E) Scheme and schedule of western blotting (arrows represent observation points). (F) Western blotting assay of NSCs examined at different time points during the protocol, with (rTMS) or without (control) rTMS treatment. Erk and p-P38 activating phosphorylation were markedly increased by rTMS but attenuated as rTMS ceased. **p < 0.01 vs. former time point. (G) Inhibition results of p-Erk1/2 and p-P38 western blotting analysis. Treatment with specific inhibitors compromises rTMS-stimulated Erk- and p-P38-activating phosphorylation in NSCs. β-actin was used as a loading control; *p < 0.05 vs. control. **p < 0.01 vs. control. ^##^*p* < 0.01 vs. rTMS group.

Using the GCBI online platform, microarray data analysis demonstrated transcriptome differences according to a select criterion (*p* < 0.05, *Q* < 0.05, fold change >1.5). The rTMS give rise to (Fig. 7a, b) remarkable alterations in genes and cell function (here, we showed the top 20 altered cell functions and the top 10 path-net in Supplementary Tables S1 and S2, respectively), and pathways affected by rTMS were generated (Fig. 7c). The outcomes demonstrated that regulation of transcription, DNA-dependent (ranked 1), transcription, DNA-dependent (ranked 2), cell cycle (ranked 3) and cell division (ranked 5), phosphorylation (ranked 12), phosphorylation protein (ranked 13), nervous system development (ranked 16) and ion transport (ranked 20) were changed after rTMS treatment. Based on KEGG and the pathways affected by rTMS, Path-net analysis revealed significantly altered pathways and their relationships. The MAPK signaling pathway, Calcium signaling pathway, TGF-beta signaling pathway, and axon guidance are significantly linked to neurogenesis (Fig. 7c). Importantly, Calcium signaling pathway, TGF-beta signaling pathway, and axon guidance have been suggested to be closely related to neurogenesis via the MAPK signaling pathway (Fig. 7d). The genes involved in those significantly altered pathways (MAPK signaling pathway, Calcium signaling pathway, TGF-beta signaling pathway, axon guidance) are listed in Supplementary Table S3. In addition, typical transcriptional factors and markers of neuronal differentiation are listed in Supplementary Table S4.

### rTMS Elevated the Phosphorylation Levels of Erk and p38

According to the bioinformatics analysis, we further investigated the classic MAPK family (Erk1/2, JNK/SAPK, p38) and their phosphorylation levels using western blotting analysis to initially explore possible mechanisms underlying the effects of rTMS on neurogenesis after ICH. The results showed that the expression levels of p-Erk1/2 and p-P38 increased with additional rTMS treatments. When the treatment was finished, the expression levels decreased in a time-dependent manner, while there were no significant changes in the expression levels of p-JNK/SAPK, JNK/SAPK, Erk1/2, and P38 (Fig. 7f). In addition, SCH772984, a specific inhibitor of Erk1/2, and SB203580, a specific inhibitor of P38, could block the phosphorylation-promoting effect of rTMS on Erk1/2 and P38 (Fig. 7g).

### rTMS Promoted Ca^2+^ Influx via Voltage-Gated Ca^2+^ Channels

**Fig. 8.**
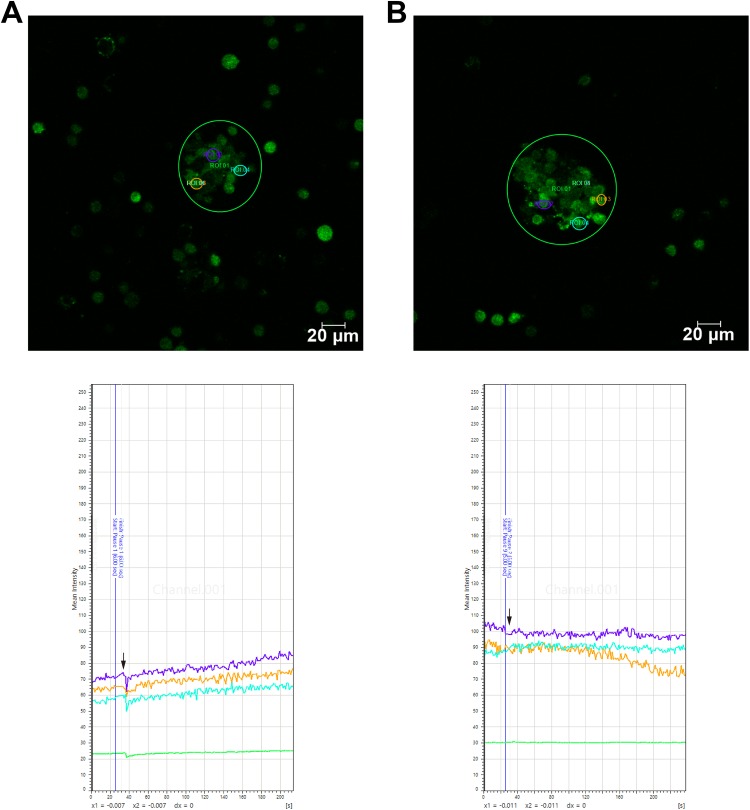
Calcium imaging of neural stem cells. Representative fields and curve graphs (the intensity of green fluorescence represents the relative level of intracellular Ca^2+^) of calcium-dye-loaded neurospheres (A) with or (B) without voltage-gated calcium ion channel-specific inhibitor nifedipine (arrows show rTMS treatment was applied), showing that rTMS treatment upregulated intracellular Ca^2+^ levels and could be blocked by nifedipine.

In light of the specific relationship between physical treatment and the MAPK signaling pathway, combined with microarray data analysis, Ca^2+^ was considered a possible initiating factor. To prove this, the intracellular Ca^2+^ level was qualified by calcium imaging. The outcome indicated that the intracellular Ca^2+^ level increased when rTMS treatment was applied (Fig. 8a) and that the effect of rTMS could be blocked by a specific voltage-gated Ca^2+^ channel inhibitor, nifedipine (Fig. 8b).

## Discussion

rTMS, a noninvasive, convenient, and physical rehabilitative tool to manipulate the intrinsic physiological processes of the body, has been used for various neurological and psychiatric diseases and for rehabilitation for therapeutic or even diagnostic purposes^13,14^. rTMS with diverse parameters produces different effects; for instance, low-frequency rTMS (<1 Hz) reduces cortical excitability and produces long-term depression, while high-frequency rTMS (>5 Hz) stimulates cortical excitability and generates long-term potentiation (LTP)-like effects^15,16^. The latest studies have demonstrated that rTMS has substantial effects on modulating proliferation and differentiation of NSCs, and give a positive prospect for refractory diseases^17^. The physical effects are always complicated: these effects are involved in many different mechanisms, including modulation of metabolism and regulation of neurotransmitters, microRNAs, and epigenetic modifications^18–20^.

NSCs are considered to be the main recovery factor after hemorrhagic stroke^21^; the dormant NSCs are activated after the damage, proliferate, and migrate to the perihematomal region^22^; and via neuronal differentiation, NSCs were observed to try to reform the functional connections among the surviving neurons after stroke and replace these damaged neural cells with limited improvement^23^. On the other hand, most NSCs around the hematoma differentiated into glial cells as a result, and aggregation of glial cells increased, which means a worsening of the inflammatory reaction. This process can give rise to a more deteriorative secondary process after hemorrhagic stroke, and the glial scar formed after hemorrhagic stroke would not aid the recovery of neurological functions^24^.

rTMS is a promising way to help recovery from ICH. Moreover, a previous study has shown that rTMS is a useful tool with unique traits that could enhance neurogenesis after ischemic stroke by up- or downregulating microRNA or activating the BDNF/TrkB signaling pathway^9–11^. To our knowledge, our study is the first to investigate the influences of rTMS on NSCs in mice after ICH. We used 10 Hz as a classic high frequency to activate NSCs^10^. Significant enhancement in neurogenesis after rTMS was observed, indicating that more NSCs in S-phase and more NSCs emerged around the hematoma. Furthermore, we found that rTMS increased the diameter and activated the renewal property of NSC spheres formed in vitro. All of the above findings were in accordance with these previous studies demonstrating that rTMS enhances neurogenesis in a central nervous system disease model or in vitro^12,25–27^. In our study, we also saw recovery, decreased neuronal damage, and vanished neurological deficits post-ICH after rTMS treatment, which is a result of the enhanced proliferation of NSCs. Therefore, we can see that rTMS may help recovery from ICH partially by mobilizing more NSCs into S-phase to produce more NPCs.

As previous research indicated, glial cell aggregation and glial differentiation of NSCs occurred in the post-ICH microenvironment, and producing more neural stem cells alone cannot assist in a functional restoration of the central nervous system after stroke^28,29^. Only the newly formed neurons connected in a functional circuit are thought to be valid, so the neuronal differentiation of NSCs is a crucial part of rehabilitation. In this study, we evaluated the influence of rTMS on the differentiation of NSCs after ICH. Earlier research has shown that an extremely low-frequency magnetic field (here referred to as 0–300 Hz) could induce neuronal differentiation of NSCs by modulating phosphorylated transcription factor cAMP response element-binding protein (CREB) and calcium flux^19,30^. In the present study, we found a remarkable neuronal differentiation induced by rTMS treatment with less aggregation of glial cells and newly formed neurons trying to reconnect the functional network. These findings are in line with the observed attenuation of brain water content and significant improvement in neurological behavioral function. Moreover, we observed a similar phenomenon in vitro, in which neural spheres differentiate into neurons rather than glial cells under an imitative pathophysiological microenvironment induced by 1% FBS. The outcomes both in vivo and in vitro were similar to a previous study, and thus it is likely that rTMS sustained the neurogenesis of NSCs and polarized them into neurons rather than glial cells. On the other hand, rTMS may suppress the potential of NSCs to become glial cells, and in vivo, we can reasonably assume that rTMS treatment may also suppress these inflammatory cells, such as microglia and astrocytes, alternatively.

To obtain more profound knowledge about the positive effects of rTMS on NSCs, further study based on data analysis showed that these related pathways, including the MAPK signaling pathway, Calcium signaling pathway, TGF-beta signaling pathway, and axon guidance, were significantly altered after rTMS. Among these pathways, Calcium signaling pathway is closely related to rTMS—the magnetic device can naturally exert effects on the ion. A previous study demonstrated that calcium signaling could promote the phosphorylation of CREB at Ser133, which subsequently recruits more histone acetyltransferase and CREB-binding protein to enhance the differentiation and transcription of neuronal genes (NeuroD1, MAP2)^19^. Using calcium imaging in a qualitative way, we found that rTMS promoted Ca^2+^ influx via voltage-gated Ca^2+^ channels. In concert with the prediction of the calcium signaling pathway made by bioinformatics analysis, Ca^2+^ accumulation will activate the calcium signaling pathway, and various abilities of this pathway explain the versatility of rTMS well. In addition, rTMS offered an initial way to launch all the processes in a more physical way. Although the link between Ca^2+^ and phosphorylation of CREB remains unclear, here, through a bioinformatics method, we can see that the MAPK signaling pathway might be the major downstream and hub signaling pathway that directly phosphorylates CREB and initiates the transcription of neuronal and proliferative genes. In the western blotting assay, we showed that phosphorylation of P38 and Erk1/2 in the classic MAPK signaling pathway could be activated by rTMS; however, phosphorylation of JNK/SAPK appeared to be minimally altered. The Erk1/2 pathway is a crucial pathway involved in cell differentiation and proliferation, and the P38 pathway is known to participate in cell differentiation when cells respond to external stress, such as electromagnetic radiation. Both of these findings are in harmony with our results. To the best of our knowledge, these data demonstrate for the first time that P38 and ERK1/2 signaling is crucial for rTMS-induced NSC proliferation and differentiation. rTMS is a particularly sophisticated physical treatment, and JNK/SAPK is also a stress-responsive pathway similar to P38, although it is mainly involved in cell death. Thus, we cannot simply draw a conclusion about the lack of effects on JNK/SAPK, so we further investigated the detailed mechanism.

From the analysis of microarray data, we also noticed that the TGF-beta signaling pathway, which plays a vital role in enhancing neuronal differentiation and reprogramming glial cells into functional neurons^31–33^, is remarkably altered and associated with the MAPK signaling pathway^34^. Inspired by these facts, we reviewed the significantly altered genes. We were surprised to find that the widely expressed transcription factors (TFs), which can independently reprogram, such as NeuroD1, Neurog2, Myt1 l and Pax6^31,35–37^, were significantly changed, although they were not ranked highly; combined with the significant alterations in the TGF-beta signaling pathway, which can enhance the reprogramming efficiency of these TFs^31–33^, these TFs will induce the reprogramming of glial cells via specific TFs. As previously described, in neurodegenerative disease and after brain damage, loss of functional neurons is often accompanied by inflammatory response, gliosis, and scarring. Using existing treatments we have great difficulties in reversing unexpected outcomes^38^. Recent studies have shown that forcing the expression of specific TFs^38–40^ or even the use of accessible small molecules^31,33^ could transform glial cells into functional neurons. If rTMS has similar effects and could be an informative tool, we confidently predict that rTMS could reprogram glial cells into neurons to some degree. Thus, we can also explain the reduction in glial cells and the increase in neurons. Interestingly, we noted that axon guidance, which is an important factor in inducing the neuron to migrate to the correct region to form functional connectivity, was also significantly altered. This finding indicated that rTMS might have the ability to promote guidance and could partially explain why rTMS improved neurobehavioral deficits so efficiently. Given these data, our transcriptome analysis suggested that the MAPK signaling pathway could be a link between proliferation and neuronal differentiation/reprogramming/ axon guidance and neurogenesis.

Our study has some potential limitations. First, rTMS is known to play an important role in neurogenesis and the induction of neuronal differentiation via regulation of Ca^2+^ CREB, BDNF pathways and microRNA^9,11,19,30^, neurotransmitters, and modulation of neuronal inhibition, excitation, connectivity, and glial cells, etc^20,41,42^. In addition, the effects of physical methods are always complex, and crosstalk among them makes the potential mechanism more complex. rTMS can be reasonably proposed to have positive effects on neurogenesis after ICH by mechanisms other than the MAPK signaling pathway analyzed bioinformatically, and more work might focus on the other approaches. Second, our study observed only the neurogenesis effects of rTMS within the acute phase (5 days) of ICH. Given that rTMS has been widely used in the rehabilitative field, the long-term outcome of rTMS after ICH should be addressed in the future. Finally, rTMS with different parameters exhibits different effects. In this study, we chose a relatively well-recognized, safe, and positive frequency of 10 Hz to determine whether other parameters, such as frequency, intensity, and sustaining time, could change the effects that need to be identified in further research; the safety of these unusual parameters should be noted as well.

## Conclusion

In summary, intrinsic NSCs are crucial for the recovery of ICH, and their proliferation and differentiation directly decide the reconstruction of post-ICH neural function. rTMS, a noninvasive, safe, and convenient tool with positive effects, is a potential therapy for experimental ICH by enhancing the neurogenesis of NSCs, guiding them to neuronal differentiation, and suppressing glial differentiation. The novel mechanism might be involved in neurogenesis, the inflammatory response, reprogramming glial cells, and axon guidance via the MAPK signaling pathway.

## Supplemental Material

supplemental_figure - Repetitive Transcranial Magnetic Stimulation Promotes Neural Stem Cell Proliferation and Differentiation after Intracerebral Hemorrhage in Mice* Click here for additional data file. supplemental_figure for Repetitive Transcranial Magnetic Stimulation Promotes Neural Stem Cell Proliferation and Differentiation after Intracerebral Hemorrhage in Mice* by Mengchu Cui, Hongfei Ge, Han Zeng, Hongxiang Yan, Le Zhang, Hua Feng and Yujie Chen in Cell Transplantation

## Supplemental Material

Supplemental_Tables - Repetitive Transcranial Magnetic Stimulation Promotes Neural Stem Cell Proliferation and Differentiation after Intracerebral Hemorrhage in Mice* Click here for additional data file. Supplemental_Tables for Repetitive Transcranial Magnetic Stimulation Promotes Neural Stem Cell Proliferation and Differentiation after Intracerebral Hemorrhage in Mice* by Mengchu Cui, Hongfei Ge, Han Zeng, Hongxiang Yan, Le Zhang, Hua Feng and Yujie Chen in Cell Transplantation
