# Supplementary figures and images for: Repetitive Transcranial Magnetic Stimulation Promotes Neural Stem Cell Proliferation and Differentiation after Intracerebral Hemorrhage in Mice*
Source: Cell Transplant. 2019 Mar 4;28(5):568–84. doi: 10.1177/0963689719834870 (PMC7103604; doi:10.1177/0963689719834870)

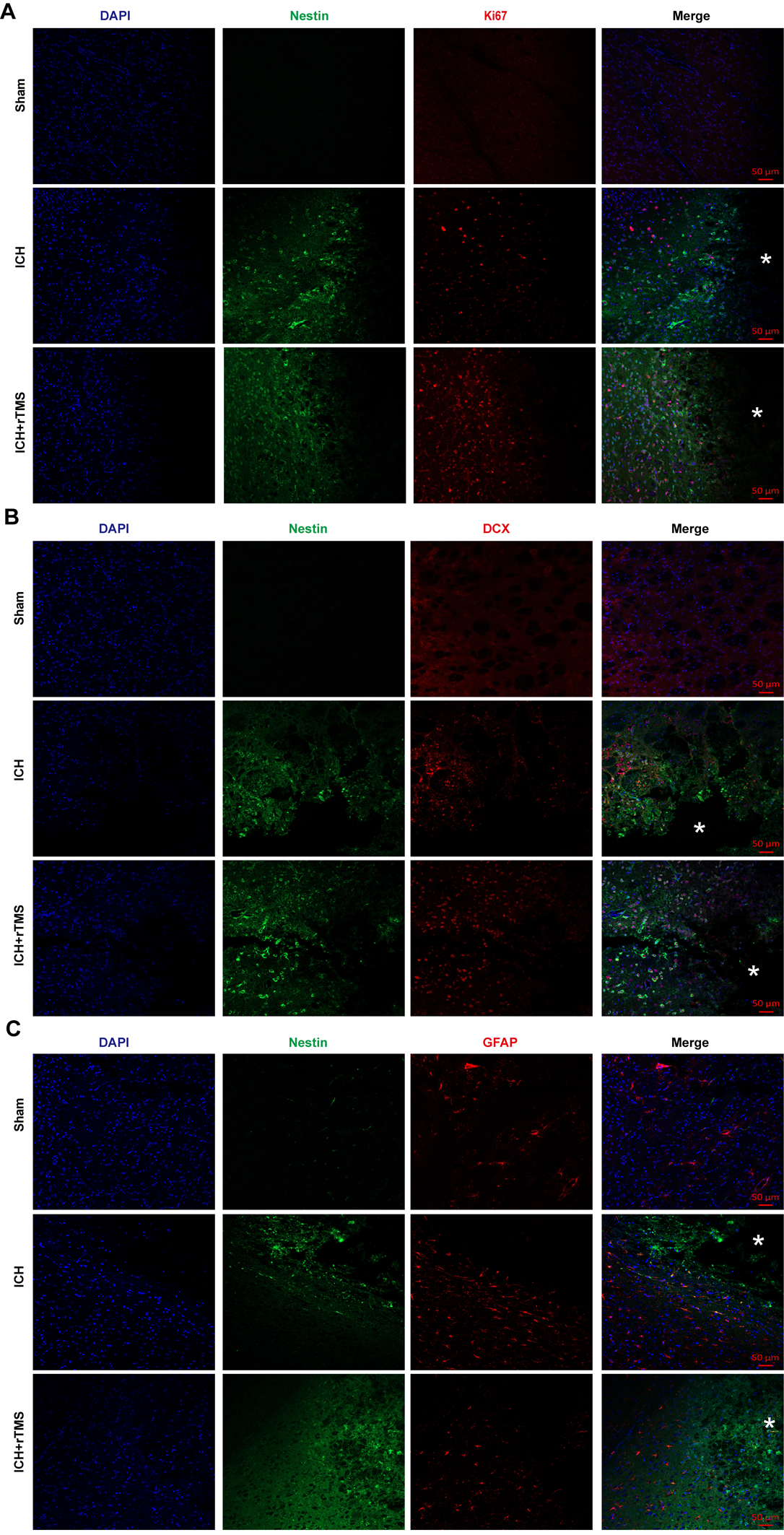

Supplement: supplemental_figure - Repetitive Transcranial Magnetic Stimulation Promotes Neural Stem Cell Proliferation and Differentiation after Intracerebral Hemorrhage in Mice* [file supplemental_figure.tif]
