# Supplementary material for: Repetitive Transcranial Magnetic Stimulation Promotes Neural Stem Cell Proliferation and Differentiation after Intracerebral Hemorrhage in Mice*
Source: Cell Transplant. 2019 Mar 4;28(5):568–84. doi: 10.1177/0963689719834870 (PMC7103604; doi:10.1177/0963689719834870)
Supplement: Supplemental_Tables - Repetitive Transcranial Magnetic Stimulation Promotes Neural Stem Cell Proliferation and Differentiation after Intracerebral Hemorrhage in Mice* [file Supplemental_Tables.pdf]

Supplementary Table S1

Rank	GO ID	GO Name	Diff Gene Counts in GO	Gene Amount in GO	Enrichment Score	p-value	FDR
1	GO:0006355	regulation of transcription, DNA-dependent	273	1852	3.696985	1.46E-79	5.41E-76
2	GO:0006351	transcription, DNA-dependent	261	1756	3.727709	9.80E-77	1.26E-73
3	GO:0007049	cell cycle	148	565	6.569606	1.02E-76	1.26E-73
4	GO:0006810	transport	257	1712	3.764917	1.89E-76	1.75E-73
5	GO:0051301	cell division	91	333	6.85367	4.47E-49	3.32E-46
6	GO:0007067	mitosis	78	241	8.11715	3.09E-48	1.91E-45
7	GO:0006974	response to DNA damage stimulus	84	361	5.835769	1.58E-39	8.40E-37
8	GO:0006260	DNA replication	51	123	10.39899	3.89E-38	1.81E-35
9	GO:0007275	multicellular organismal development	131	954	3.443888	1.09E-34	4.50E-32
10	GO:0006281	DNA repair	70	291	6.032969	4.29E-34	1.59E-31
11	GO:0007155	cell adhesion	91	500	4.564544	1.02E-33	3.43E-31
12	GO:0016310	phosphorylation	105	684	3.849987	4.38E-32	1.36E-29
13	GO:0006468	protein phosphorylation	97	611	3.98159	6.80E-31	1.94E-28
14	GO:0008152	metabolic process	124	991	3.138153	5.53E-29	1.47E-26
15	GO:0008150	biological_process	620	10237	1.518955	1.06E-28	2.63E-26
16	GO:0007399	nervous system development	65	307	5.310079	2.77E-28	6.43E-26
17	GO:0006397	mRNA processing	57	278	5.142284	3.35E-24	7.31E-22
18	GO:0015031	protein transport	80	538	3.729355	9.15E-24	1.89E-21
19	GO:0043066	negative regulation of apoptotic process	75	480	3.918737	1.11E-23	2.16E-21
20	GO:0006811	ion transport	81	570	3.563988	9.38E-23	1.74E-20

Supplementary Table S2

Rank	Pathway ID	Pathway Name	Outdegree	Indegree	Degree
1	4010	MAPK signaling pathway	5	31	36
2	5200	Pathways in cancer	26	0	26
3	4210	Apoptosis	2	23	25
4	4110	Cell cycle	3	21	24
5	4115	p53 signaling pathway	2	18	20
6	4020	Calcium signaling pathway	5	11	16
7	4310	Wnt signaling pathway	7	8	15
8	4012	ErbB signaling pathway	8	6	14
9	4510	Focal adhesion	7	7	14
10	4350	TGF-beta signaling pathway	4	8	12

Supplementary Table S3

Pathway ID	Pathway Name	Diff Gene Counts in	Gene Amount in Pathway	Enrichment Score	p-value	FDR	Gene Symbols
4010	MAPK signaling pathway	35	259	3.38917751444749	6.953329573035 85E-10	5.63459465401181E-09	|Ppp5c|Cacna1b|Cacna1e|Cacna2d1|Cacna2d3|Taok1|Ppp3ca|Cdc25b|Hras1|Atf4|Fgf14|Mapkapk2|Mapk10|Atf2|Rasa2|Flnb|Mapk8|Pdgfra|Chuk|Ddit3|Fgf21|Gadd45a|Map3k2|Rps6ka3|Braf|Stk3|Tgfb2|Mef2c|Tgfbr1|Cacnb4|Cacng2|Map4k3|Rasa1|Rps6ka5|Dusp6|
4360	Axon guidance	31	133	5.84569414897936	2.46808249836573E-15	8.28570553022782E-14	|Nfatc1|Sema3a|Epha6|Unc5d|Hras1|Pak7|Nfat5|Rasa1|Epha5|L1cam|Ntng1|Unc5c|Robo1|Slit2|Dcc|Epha4|Ntn4|Epha7|Met|Rock2|Epha3|Plxna4|Plxna3|Sema5a|Robo2|Ppp3ca|Lrrc4c|Ntn1|Rock1|Nrp1|Pak3|
4020	Calcium signaling pathway	16	185	2.1690736092464	0.0074160538165082	0.0145231053906619	|Pdgfra|Itpkb|Ppp3ca|Plcb4|Erbb4|Cacna1e|P2rx3|Plcb1|Slc8a1|Ednra|Cacna1b|Slc25a5|Pde1a|Atp2b1|Ryr3|Plcd4|
4350	TGF-beta signaling pathway	15	83	4.53251450727315	1.84122661303045E-06	9.20613306515224E-06	|Thbs1|Inhba|Tgfbr1|Rock2|Zfyve16|Id3|Rock1|Rps6kb1|Bmpr2|Bmp4|Bmp2|Tgfb2|Id1|Dcn|Acvr2a|

Supplementary Table S4

Probe Set ID	Gene Symbol	Accession Number	Database Source	Mean Signal of Group rTMS	Mean Signal of Group Blank	d Score	Fold Change	p-value	q-value	Gene Feature
10484276	Neurod1	NM_010894	RefSeq	5.922978	4.717428	7.560285	2.306252	0.000263	0	up
10607156	Dcx	NM_001110222	RefSeq	9.798409	8.495217	4.101576	2.467743	0.002731	0	up
10495964	Neurog2	NM_009718	RefSeq	6.745101	6.085297	6.464297	1.579869	0.000512	0	up
10474312	Pax6	NM_001244198	RefSeq	7.508712	7.09887	2.988355	1.328541	0.006178	8.80E-05	up
10347036	Map2	NM_001039934	RefSeq	9.755172	8.989564	3.065041	1.700086	0.005817	8.80E-05	up
10395074	Myt1l	NM_008666	RefSeq	7.953199	7.128191	4.299004	1.771545	0.00236	0	up
